# Movement control exercise versus general exercise to reduce disability in patients with low back pain and movement control impairment. A randomised controlled trial

**DOI:** 10.1186/1471-2474-12-207

**Published:** 2011-09-23

**Authors:** Jeannette Saner, Jan Kool, Rob A de Bie, Judith M Sieben, Hannu Luomajoki

**Affiliations:** 1 Institute of Physiotherapy, School of Health Professions, Zurich University of Applied Sciences, Technikumstrasse 71, Postfach, CH - 8401 Winterthur, Switzerland; 2 Dept. of Methodology and Statistics, CAPHRI School of Public Health and Primary Care, Maastricht University, Maastricht, The Netherlands; 3 Department of Anatomy & Embryology, Maastricht University, Maastricht, The Netherlands

## Abstract

**Background:**

Non-specific low back pain (NSLBP) in subacute and chronic stages can be treated effectively with exercise therapy. Research guidelines recommend evaluating different treatments in defined subgroups of patients with NSLBP. A subgroup of patients with movement control impairment (MCI) improved significantly on patient specific function and disability in a previous case series after movement control exercises.

**Methods/Design:**

In a randomised controlled trial (RCT) we will compare the effectiveness of movement control and general exercise in patients with MCI. 106 participants aged 18 - 75 will be recruited in 5 outpatient hospital departments and 7 private practices.

Patients randomly assigned to the movement control exercise group will be instructed to perform exercises according to their MCI. The general exercise group will follow an exercise protocol aimed at improving endurance and flexibility. Patients in both groups will receive 9 - 18 treatments and will be instructed to do additional exercises at home.

The primary outcome is the level of disability assessed using the patient specific functional scale (PSFS) which links the perceived pain to functional situations and is measured before treatment and at 6 and 12 months follow-up. Secondary outcomes concern low back pain related disability (Roland Morris questionnaire, RMQ), graded chronic pain scale (GCPS), range of motion and tactile acuity.

**Discussion:**

To our knowledge this study will be the first to compare two exercise programs for a specific subgroup of patients with NSLBP and MCI. Results of this study will provide insight into the effectiveness of movement control exercise and contribute to our understanding of the mechanisms behind MCI and its relation to NSLBP.

**Trial registration:**

Current Controlled Trials ISRCTN80064281

## Background

Low Back Pain (LBP) is one of the major concerns of current health care [1-5]. Only 10% of LBP cases can be attributed to specific disorders like nerve root compression, vertebral fracture, tumour, infection, inflammatory diseases, spondylolisthesis or spinal stenosis. Consequently, NSLBP, in which the cause of symptoms is unknown, is diagnosed in about 90% of all patients and is a health problem of high economic importance [6].

Patients with NSLBP present diverse clinical findings, courses of disease and prognoses. They therefore make up a heterogeneous group of patients, which may explain why treatment effects in numerous studies looking at specific physiotherapy treatments in the NSLBP group are often discouraging. Identifying defined subgroups of patients within the NSLBP population has been a major focus in recent research [7-12]. Current European guidelines encourage outcome studies in subgroups of patients with a shared diagnostic pattern or prognosis that might benefit from specific treatments [1]. This research agenda is expected to reveal further evidence for the effect of treatments designed for specific subgroups [13]. Of 767 RCTs about the effect of conservative treatment on chronic LBP performed and published between 1982 and 2008, 68 publications examined manual or exercise therapy, of which five studies had an additional subclassification and matched treatments [14].

There is no evidence for the effectiveness of exercise in patients with acute LBP of a less than 6 week duration [1,15-17], but exercise therapy is effective in chronic and subacute LBP [16]. There is evidence that home exercise may be effective in decreasing pain and disability, but results have shown no significant difference between exercise types on work disability [1,18]. There is moderate evidence suggesting that exercise therapy may prevent recurrences of LBP, but there is no evidence for a difference in effect between types of exercise [19]. Individually designed exercise programs are recommended [17] but the question remains as to which types of exercise are effective for which subgroups of patients.

Within the framework of a new NSLBP classification system developed by O'Sullivan, one of the subgroups of patients that can be distinguished contains those suffering from MCI [11]. In a first step, this classification of NSLBP distinguishes between patients with non-mechanical disorders, and patients with mechanical disorders. Whereas in patients with non-mechanical NSLBP, psychosocial factors, fear and catastrophising play central roles, pain in relation to posture and movement is predominant in patients with mechanical NSLBP. Patients with mechanical NSLBP are further divided into those with movement impairment (MI) and movement control impairment (MCI). Patients with MI may suffer movement restrictions in single or multiple directions. MCI is defined as a deficit in the control of movements during functional daily activities. The range of the movement is not restricted in the MCI group.

Clinical tests to identify MCI were developed in recent research [11,20-26]. Further evaluation revealed six tests which reliably detect MCI in patients [27]. (Additional files 1) These tests will be used to select patients with MCI for this study. Movement control tests are easy to perform in clinical practice. Tests and clinical presentation allow a further classification of movement control dysfunctions according to direction in extension, flexion, frontal plane and multi-directional MCIs. (Table 1)

**Table 1:** Five distinct directional patterns of movement control impairment [72] (personal communication)

Direction of movement control impairment	Pain aggravation	Pain relief	Movement control deficit
**Flexion**	Sustained flexion of lumbar spine, e.g. when sitting	Extension of lumbar spine, e.g. when standing and walking	Difficulty controlling lordosis in sitting and flexed positions

**Active Extension**	Sustained extension of lumbar spine	Flexion of lumbar spine, relaxing in flexed posture. Breathing exercises	Difficulty flexing when sitting or breathing with diaphragm

**Passive extension**	Extension of lumbar spine, e.g. when standing or walking slowly	Flexion of lumbar spine, e.g. while sitting	Tilting pelvis posteriorly

**Frontal pain control**	unilateral pain in unilateral loading and sidebending	Control pelvis and thorax in frontal plain	Maintain symmetric posture

**Multidirectional pattern**	Multidirectional	Changing lumbar spine position	Difficulty assuming neutral lordotic spinal positions

Description: Specification of main symptoms and signs to classify the direction of movement control impairment

Two mechanisms are proposed to explain the impaired movement control behaviour. One relates to conditioning and habituation, which are important factors in motor learning. Patients use postures and movements that are potentially harmful due to maladaptive processes, like avoidance or overuse during the acute pain phase [28]. Another mechanism is non-awareness of the posture's pain provocation. Altered cortical representation of the lumbar spine in the presence of pain may play an important role [29]. Two point discrimination is decreased in patients with NSLBP, indicating changes in cortical representation because of NSLBP [30]. Both mechanisms can either be induced by pain or be the cause of pain.

It is hypothesised that, once movement control is impaired, it results in repetitive mechanical deformation of innervated tissue and leads to increased nociceptive input to the central nervous system and, therefore, pain [31]. All joint capsules, ligaments, tendons and muscles are possible pain sources, especially due to continuous strain or longstanding repetitive movements [31,32]. Repeated misuse of these tissues can also initiate the inflammatory cascade, a further cause of pain [33].

The inter-rater reliability of the clinical classification of NSLBP in MI and MCI is very high; in experts and raters with less experience, k = 0.85 and k = 0.6 respectively [34,35]. The inter- and intra-rater reliability of the previously described 6 active tests performed by the patient was evaluated as good to substantial [27]. Validity of MCI tests is supported by significantly different results in healthy subjects, with 0.75 positive MCI tests (95% CI 0.55-0.95) and low back pain patients with 2.21 positive MCI tests (95%CI 1.94-2.48, effect size d = 1.18) [36].

In a preceding prior controlled case series, 38 preselected patients with positive MCI tests were treated with an individualised movement control exercise program and showed improvements in MCI test performance associated with improvements in patient specific functional complaints and disability [37]. The present study will compare movement control training with general exercise in a randomised controlled trial.

MCI exercises are often and falsely referred to as motor control exercises, spinal stabilisation or core stability exercises. While MCI exercises aim to improve function through repetitive normal use, the latter retrain delayed muscle activity first in order to improve control of the spine. Several trials and systematic reviews have evaluated the effect of stabilising exercises with conflicting results [38-42]. A recent randomised placebo-controlled trial with a specific stabilisation program in a population with chronic low back pain found a significant improvement in activity in both the short and long term when measured with a patient specific functional scale (PSFS), and a short term improvement for global impression of recovery and disability [43]. However, they did not subgroup the patients and the effects were not beyond the smallest minimal meaningful clinical change.

The general exercise treatment is based on treatments used in previous studies. Exercises were developed to improve endurance, strength and flexibility of the spinal tissues [44]. Experimental studies have shown that these exercises involve many global and local muscles and improve the stability of the spine [45,46]. In an RCT of patients with subacute and chronic NSLBP, a course of only general exercise was compared with a combination of general exercise and stabilisation exercise. Immediately after treatment, disability was significantly lower in the general exercise group. No differences were found in other outcomes or at follow-up time points [47].

Psychosocial factors have an important impact on treatment outcome in NSLBP patients who also show non-acute [48] depression, pain catastrophising, fear of pain and avoidance. These aspects are part of the fear-avoidance model of chronic pain, and previous data has reported its various effects on treatment outcome [49]. Pain-related fear and subsequent avoidance of movements that patients believe to be harmful can also lead to disability and physical deconditioning [50]. In our study patients with a high risk of psychosocial problems are excluded based on an assessment with the Örebrö Musculoskeletal Pain Questionnaire (ÖMPQ) [51,52]. This influential variable will be observed using the Fear-avoidance beliefs questionnaire.

This randomised controlled trial will, to our knowledge, be the first to compare two exercise programs for a well-defined subgroup of patients with non-specific low back pain and movement control impairment. We will evaluate the effect of individualised movement control exercise versus general exercise on disability during a one year follow-up period.

## Methods/Design

### Design

**Figure 1:**
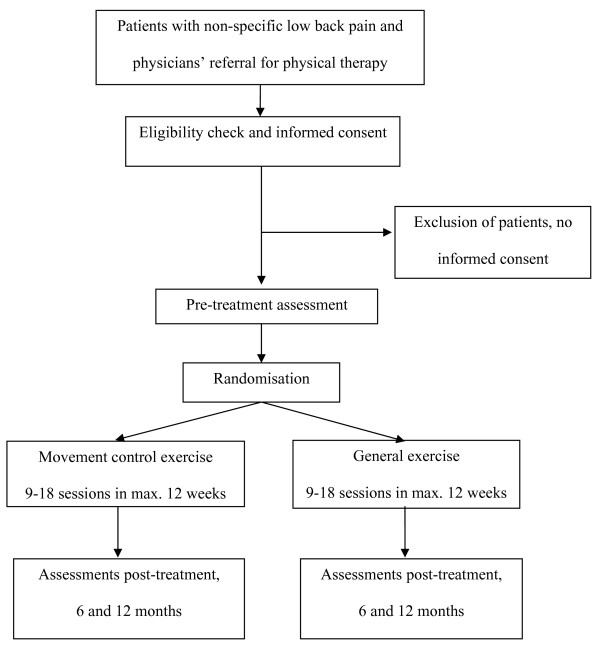
**Flow-chart of research design**.

In this randomised controlled trial we will include patients with non-acute NSLBP and MCI. Patients will be recruited and treated in 5 hospital outpatient departments and 8 private practices in Switzerland. Movement control exercise will be compared to general exercise. Treatment outcomes are to be measured at baseline, post-treatment, and at 6 and 12 months follow-up (Figure 1).

Ethical approval has been granted by the Swiss Ethics Commission for Clinical Trials in the cantons of Zurich, Basel and Aargau (Switzerland). Reporting of the RCT will follow the recommendations of CONSORT Statement 2010 http://www.consort-statement.org [53].

### Hypotheses

1. In NSLBP patients with MCI, treatment with movement control exercises will result in a significant decrease in disability compared to general exercise, measured with the patient specific functional scale.

2. In NSLBP patients with MCI, improvement in motor control of the lumbar spine, assessed with standardised clinical tests, is associated with functional improvement and pain reduction.

### Participants and recruitment

Admission criteria for the study are described in Table 2. Eligible are patients with NSLBP referred to physiotherapy by their physician. Patients will be recruited by the participating physiotherapists. Patients are included in the study if at least 2 MCI tests are positive and if they present clinical symptoms of MCI as described by [21,24,25,27,30,36]. Patients with specific LBP, identified by the physician or by the physiotherapist are excluded from the study. To prevent floor effects in outcome measurement, a minimal disability of 5 points on the Roland and Morris Disability Questionnaire (RMQ) [54] is required. Excluded are patients with predominant psychosocial factors, defined as a score of more than 130 points on the Örebrö Musculoskeletal Pain Questionnaire (ÖMPQ) [51,52]. An ÖMPQ score of over 130 has been shown to correctly predict failure of return to work in 86% of cases [51]. The German version of the ÖMPQ has been used in a previous study in Switzerland [55].

**Table 2:** Inclusion and exclusion criteria

Inclusion criteria	- Age 18 to 75 years - Non-acute LBP (> 6 weeks duration of symptoms) and less than 3 months of sick leave due to LBP - Two or more positive tests for impaired movement control (Luomajoki et al 2007) - At least 5 points on Roland Morris Disability questionnaire [54] - Clinical behaviour: posture and movement aggravate and ease symptoms; symptoms are relieved by reducing the strain on the lumbar region - Written informed consent
Exclusion criteria	- Specific LBP (Fractures, carcinoma, anomalies, nerve root affection with neurological signs e.g. sensitivity or reflex loss, muscle weakness, radicular pain below the knee) - Less than 6 weeks post-surgery following all surgery on the lower back - post-surgery with spondylodesis - high level of psychosocial risk factors (> 130 points on the ÖMPQ) - Peripheral or central neurological disease - Contraindications for exercise, e.g. major cardiovascular disease or postural hypotension - Inability to understand the purpose of the study - Psychological or psychiatric problems - Chronic abuse of toxic substances such as drugs or alcohol - Use of neuroleptics, sedatives, anti-epileptics and antidepressants

### Baseline assessment and randomisation

After eligibility has been confirmed, patients will be informed about the study comparing two treatments that are widely used in physiotherapy. After obtaining written informed consent, baseline measurements will be performed (see next paragraph). Participants will then be randomised using block allocation with a block size of four, to receive either movement control or general exercise. Randomisation is concealed and performed by an independent assistant at the School of Health Professions at the Zurich University of Applied Sciences via telephone.

### Outcome measurements

Criteria for the selection of the outcome measurements are reliability, validity and sensitivity for statistical change.

#### Primary outcome

**Patient-specific LBP-related disability **will be assessed using the patient specific functional scale (PSFS), a self-reported measurement for up to three individual activity limitations rated on an 11-point numeric rating scale ranging 0-10 [56]. Measurement is taken at baseline, post-treatment, and at 6 and 12 months follow-up. Reliability and validity have been reported to be good [57,58]. Internal and external responsiveness are good, indicating that the test detects a change in active limitation and that this change is meaningful [59]. In patients with low levels of activity limitations, the PSFS has better responsiveness than the RMQ [60].

#### Secondary outcomes

**General LBP-related disability **will be assessed with the RMQ [54]. It consists of 24 dichotomous questions to be answered with yes or no, and has a maximum disability score of 24 points. Measurement is taken at baseline, post-treatment and at 6 and 12 months follow-up. Reliability and validity have been widely tested, reliability is high, and construct and internal validity are good [61]. The reliability and validity of the German version have been confirmed [48].

**Pain, daily activities, social participation and professional participation **will be assessed using the Graded Chronic Pain Scale (chronic pain grade = CPG) [62]. The German version of the questionnaire shows significant correlations with other assessments of disability and staging of chronic pain, and a good internal consistency (Cronbach's alpha = .82) [63]. Measurement is taken at baseline, post-treatment, and at 6 and 12 months follow-up. Sports and leisure activities will be assessed with a self-administered questionnaire developed for this study.

**Range of motion **is measured pre- and post-treatment by finger to floor distance, a valid, reliable and responsive measurement correlating with radiography in patients with chronic low back pain [64].

**Tactile acuity (two point discrimination TPD) **is measured in the paravertebral lumbar region using a plastic caliper ruler between L1 and the iliac crest. Measurements are taken horizontally and vertically. The two point discrimination threshold is where the smallest distance at which the patient correctly reports feeling two points of the caliper instead of one is located. We will calculate the average of two procedures; one starting with an extended position of the calipers with the distance being decreased, the other starting with calipers in a contracted position and the distance being increased. Out of sequence tests are performed to avoid the recognition of a pattern. This test was recently used in patients with NSLBP [30]. Reliability was not formally evaluated in this population. TPD is measured pre- and post-treatment.

**Direct and indirect LBP related costs **will be calculated based on the use of medication and medical treatment. Data are recorded according to information given by the patient. Indirect costs are calculated in sick leave days costs based on Swiss average salary data (Statistisches Jahrbuch der Schweiz, 2011).

### Treatment effect modifiers

The following relevant covariates will be recorded in order to allow them to be controlled for in the analysis of this study. Movement control and endurance are addressed in the two treatment groups and are expected to modify treatment effect.

**Movement control impairment **of the lumbar spine is assessed using 6 tests described in the backgrounds section [65]. Post-treatment the test will be recorded on videotape. The evaluation of video footage will be carried out by a specially trained physiotherapist who will be blinded to the treatment allocation and data from previous measurements.

**Endurance of lumbar and abdominal muscles **is assessed using static isometric strength tests for trunk extension and for trunk flexion pre- and post-treatment [66,62].

**Fear avoidance beliefs **will be measured with the self-report Fear-avoidance beliefs questionnaire for physical activities and work developed by Waddell et al [67,68]. The validated Swiss German Version will be used, allowing prediction of treatment outcome [67] It will be administered at baseline.

Patient's regular use of **home exercises **will be assessed at 6 and 12 months follow-up.

**Personal characteristics **(age, gender, previous episodes of back pain) will be collected at baseline.

### Interventions

Assessors and treating physiotherapists in both groups are trained for at least 4 hours and receive a manual containing descriptions of procedures and checklists. To support adherence to the treatment procedures, a structured recording form is provided. This study uses a pragmatic approach to treatment progression in both intervention groups. The therapist responsible for treatment has to select from exercises permitted for the relevant treatment group. The exercise prescription for the individual patient is determined by the clinical judgement of the therapist. Patients in each group will be treated by their specially trained physiotherapists in individual 30 minute sessions. Patients will receive 9-18 treatments within a period of 12 weeks. The number of treatments will be recorded. At least 20 minutes of each session are to be used for exercise according to the protocol. If required, a maximum of 10 minutes can be used for other physical therapy applications. Therapy will be monitored and evaluated using a therapists' treatment diary.

Patients are instructed to do at least three home exercises of either movement control or general exercise. They are strongly encouraged to continue them during the follow-up year. Patients are informed about frequency, number of repetitions and the intensity at which they are to perform the exercises.

#### Movement control exercise group

The patients in the movement control group will receive exercise treatment aimed at improving movement control of the lumbar spine as described in previous publications [21,34,69]. Patient education addresses awareness of positive and negative postural and movement related behaviour and increasing self-efficacy. Exercises are selected based on the direction of the impairment i.e. flexion, extension or frontal plane. The first step for patients is to learn to control the position and movement of the lumbar spine in different postures such as standing, squatting, 4 point kneeling and sitting. Movement control is practiced in combination with upper and lower extremity movements. In a second step, the difficulty level of exercise is increased through additional loading using long leavers or weights. If necessary, stretching/lengthening is applied after movement control has improved. Sports and strength training are allowed once good movement control is achieved.

#### General exercise group

Patients in this group are to be treated with the aim of improving endurance, strength and flexibility of the lumbar region. Patient education will address the importance of exercise and strength to reduce LBP. Exercises will address abdominals, erector spinae, gluteal, quadriceps and hamstrings muscle groups. The standardised exercise program starts in non-weight bearing positions and can be progressed by increasing load. Weights and resistance will be individually and progressively increased according to the guidelines of the American College of Sports Medicine [70]. The use of equipment is not standardised and will be left to the discretion of the therapists.

#### Main treatment contrast

The main difference between the two treatments is the instruction of movement control in the MCI group, which will not be applied in the general exercise group.

### Sample size calculation

Based on the results of our previous case series, inter-group difference in improvement of 0.9 points on the PSFS is to be used [71]. This difference is also clinically relevant as it displays an improvement of approximately 20% compared with an expected PSFS baseline score of 5 points. We used a standard deviation of 1.5 points, alpha was set at 0.05 and the power was set at 0.9. In each group, 48 patients are needed. Anticipating a 10% drop-out rate, the required sample size was set at 106 patients to be randomised.

### Data analysis

The comparability of both groups on prognostic and outcome variables at baseline will be analysed using two-sample t-tests in data with a normal distribution, Wilcoxon tests in non-parametric data and Chi-square tests in nominal data. An intention-to-treat analysis will be utilised in which all participants will be analysed in the group to which they were originally assigned. Differences between the groups over time are measured by Mann-Whitney-U-test. The influence of baseline differences on outcome measurements will be assessed in a multivariable linear regression analysis. A regression analysis of the factors being positive or negative predictors will be conducted based on covariates measured at baseline. Statistical significance is set at p < 0.05.

### Blinding

Patients and therapists cannot be blinded to treatment. To keep patients unaware of any expected treatment group benefit, patients will be informed that the effect of two well-established therapies is to be evaluated. An independent and blinded assessor will record videos of the movement control tests at the end of the treatment phase and perform the post-treatment physical examination. A second blinded assessor will rate the video recordings of the MCI tests. Statistical analysis will be blinded regarding treatment group code. The researcher who will perform the statistical analyses will not be involved in taking the measurements.

## Results

Inclusion of patients began in July 2010 and is expected to last until the end of 2011. Results are expected in 2013.

## Discussion

This randomised controlled trial will compare the effectiveness of two exercise-based physiotherapy treatment protocols in a well-defined subgroup of NSLP. Inclusion is based on the clinical diagnosis of MCI with clinical tests shown to be reliable. The MCI tests allow easily applicable selection of participants. We will evaluate whether a treatment protocol addressing movement control problems is more beneficial than a general exercise protocol.

The multicentre design of the study allows treatment by different physiotherapists and improves generalisability. The selection of patients reflects the population usually found in different clinical settings. Randomisation is organised centrally and prevents selection bias.

Both treatment protocols are widely used and well-established in physiotherapy. The physiotherapists treating each group are equally instructed and experienced in applying the respective treatments. This prevents a disadvantage for the participating patients regarding group allocation.

Blinding the therapists is generally not possible. To minimise measurement bias, the measurements taken after the treatment will be taken by an assessor not involved in the treatment procedures.

The results of this study will provide evidence to improve the selection of exercise treatments for patients with NSLBP and MCI. Results will also contribute to the understanding of the mechanisms behind MCI and its relation to low back pain.

## Abbreviations

CPG: Chronic pain grade; GCPS: Graded chronic pain scale; MCI: Movement control impairment; MI: Movement impairment; NSLBP: Non-specific low back pain; ÖMPQ: Örebrö musculoskeletal pain questionnaire; PSFS: Patient specific functional scale; RCT: Randomised controlled trial; RMQ: Roland Morris disability questionnaire; TPD: Two point discrimination

## Competing interests

The authors declare that they have no competing interests.

## Authors' contributions

HL originated the idea of the study, which is based on his previous work. HL, JK, JS, JMS designed the trial protocol. JS drafted the manuscript and the other authors revised it critically, corrected draft versions and approved the final manuscript.

## Pre-publication history

The pre-publication history for this paper can be accessed here:

http://www.biomedcentral.com/1471-2474/12/207/prepub

## Supplementary Material

Additional file 1 **Assessments for movement control**. Six tests which are instructed twice and demonstrated the third time. If two out of six tests are not correctly performed a movement control impairment can be diagnosed. Click here for file
