# Supplementary material for: Movement control exercise versus general exercise to reduce disability in patients with low back pain and movement control impairment. A randomised controlled trial
Source: BMC Musculoskelet Disord. 2011 Sep 23;12:207. doi: 10.1186/1471-2474-12-207 (PMC3189916; doi:10.1186/1471-2474-12-207)
Supplement: Additional file 1 — Assessments for movement control. Six tests which are instructed twice and demonstrated the third time. If two out of six tests are not correctly performed a movement control impairment can be diagnosed. [file 1471-2474-12-207-S1.PDF]

Additional files

Test	Correct performance	Movement dysfunction
**Figure 1, Test 1: "Waiters bow":**<br>Flexion of the hips in upright standing position without movement (flexion) of the low back	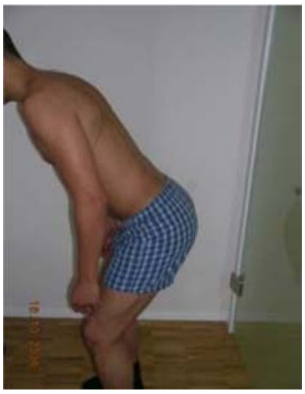<br>Forward bending of the hips without movement of the low back (50-70° Flexion hips).	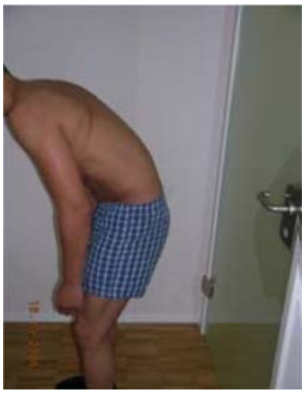<br>Angle hip Flexion without low back movement less than 50° or Flexion occurring in the low back.
**Figure 2, Test 2: Pelvic tilt**<br>Active dorsal tilt of pelvis in upright standing	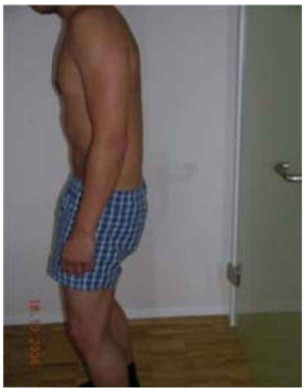<br>Actively in upright standing; keeping thoracic spine in neutral, lumbar spine moves towards Flexion.	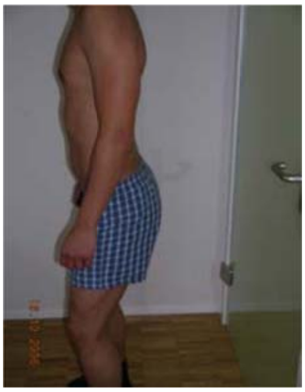<br>Pelvis does not tilt or low back moves towards Extension or compensatory Flexion in thoracic spine.
**Figure 3, Test 3. One leg stance**<br>From normal standing to one leg stance: measurement of lateral movement of the belly button. (Position: feet one third of trochanter distance apart).	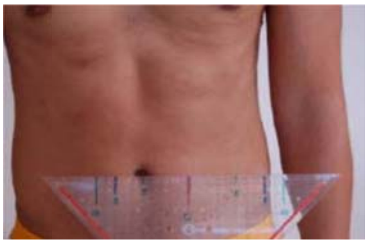<br>The distance of the transfer is symmetrical right and left. Not more than 2 cm difference between sides.	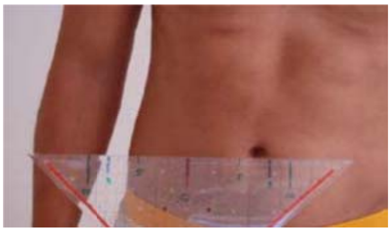<br>Lateral transfer of belly button more than 10 cm. Difference between sides more than 2 cm.

Figure 4, Test 4: Sitting knee extension.

Upright sitting with neutral lumbar lordosis; extension of the knee without movement (flexion) of low back

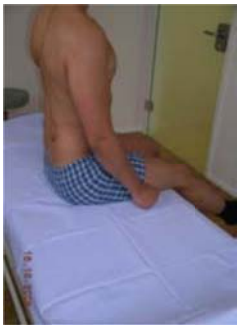

Upright sitting with neutral lumbar lordosis; extension of the knee without movement of low back (30-50° Extension of the knee is normal).

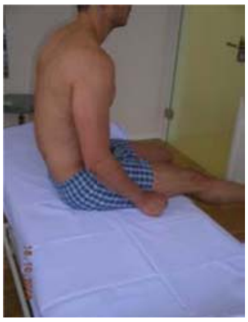

Low back is moving in flexion. Patient is not aware of the movement of the back.

Figure 5, Test 5: Rocking forward/ backward.

Quadruped position. Starting position 90° hip flexion. Transfer of the pelvis backwards and forwards ("rocking") keeping low back in neutral.

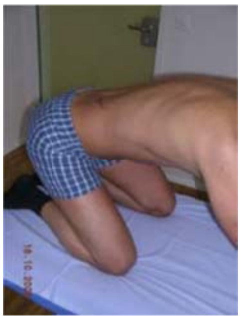

120° of hip flexion without movement of the low back by transferring pelvis backwards.

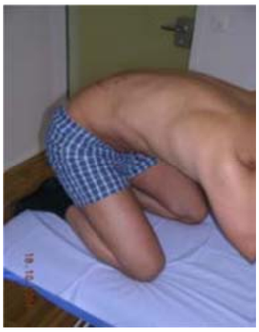

Hip flexion causes flexion in the lumbar spine (typically the patient is not aware of this).

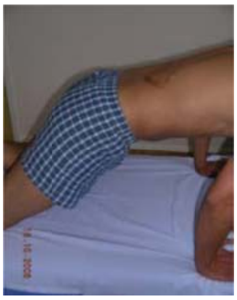

Rocking forwards to 60° hip flexion without movement of the low back

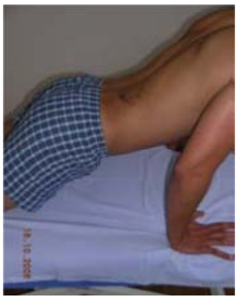

Hip movement leads to extension of the low back

Figure 6, Test 6: Prone knee flexion

Prone lying, active knee Flexion

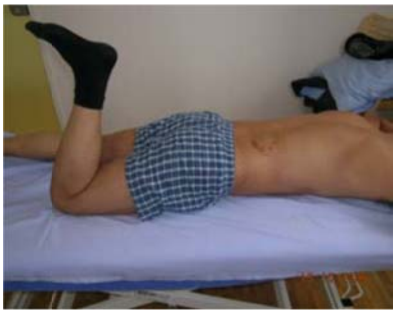

Active knee flexion at least 90° without movement of the low back and pelvis.

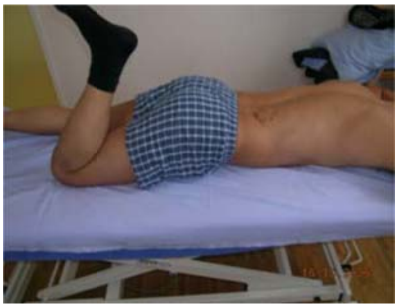

By the knee flexion low back does not stay neutral maintained but moves in extension or rotation

Additional file 1 – Assessments for movement control

Six tests which are instructed twice and demonstrated the third time. If two out of six tests are not correctly performed a movement control impairment can be diagnosed.
